# Macrovascular and renal microvascular complications in West Africans with intermediate hyperglycemia living in West Africa and Europe: The RODAM study

**DOI:** 10.1016/j.heliyon.2023.e19334

**Published:** 2023-08-20

**Authors:** Emmanuel Bannerman-Williams, Charles F. Hayfron-Benjamin, Yacoba Atiase, Silver Bahendeka, Karlijn Meeks, Kerstin Klipstein-Grobusch, Juliet Addo, Frank Mockenhaupt, Matthias B. Schulze, Erik Beune, Bert-Jan van den Born, Charles Agyemang

**Affiliations:** a Department of Public and Occupational Health, Amsterdam UMC, University of Amsterdam, Amsterdam Public Health Research Institute, Amsterdam, the Netherlands; b Department of Internal and Vascular Medicine, Amsterdam UMC, University of Amsterdam, Cardiovascular Sciences, Amsterdam, the Netherlands; c Department of Surgery, University of Ghana Medical School, Ghana; d Department of Physiology, University of Ghana Medical School, Ghana; e Department of Anaesthesia and Critical Care, University of Ghana Medical School, Ghana; f Department of Medicine and Therapeutics, University of Ghana Medical School, Ghana; g MKPGMS-Uganda Martyrs University, Kampala, Uganda; h Division of Endocrinology, Diabetes, and Metabolism, Department of Medicine, Johns Hopkins University School of Medicine, Baltimore, MD, USA; i Center for Research on Genomics and Global Health, National Human Genome Research Institute, National Institutes of Health, Bethesda, MD, USA; j Julius Global Health, Julius Center for Health Sciences and Primary Care, University Medical Center Utrecht, Utrecht University, the Netherlands; k Division of Epidemiology and Biostatistics, School of Public Health, Faculty of Health Sciences, University of the Witwatersrand, Johannesburg, South Africa; l Department of Non-Communicable Disease Epidemiology, London School of Hygiene and Tropical Medicine, London, UK; m Institute of Tropical Medicine and International Health, Charité Universitätsmedizin Berlin, Berlin, Germany; n Department of Molecular Epidemiology, German Institute for Human Nutrition Potsdam-Rehbruecke, Nuthetal, Germany; o German Center for Diabetes Research (DZD), Neuherberg, Germany; p Institute of Nutritional Science, University of Potsdam, Nuthetal, Germany

**Keywords:** Intermediate hyperglycemia, Prediabetes, Diabetes complications, Microvascular complications, Macrovascular complications, Sub-saharan africa, RODAM study

## Abstract

**Background:**

Metabolic conditions, including intermediate hyperglycemia (IH), affect migrants to a greater extent than the populations of origin. Evidence suggests that IH increases the risk of vascular complications, but it is unclear whether the differences in IH between the non-migrant and migrant populations translate to differences in vascular complications between the two populations. We compared the prevalence of macrovascular and renal microvascular complications among West Africans with IH living in West Africa and their migrant compatriots in Europe.

**Methods:**

Data from the multicenter Research on Obesity and Diabetes among African Migrants(RODAM) study were analyzed. Ghanaians with IH(524 non-migrant and 1439 migrants) were included. Logistic regression analyses were used to determine the associations between migrant status and macrovascular [coronary artery disease(CAD) and peripheral artery disease(PAD)] and renal microvascular[nephropathy] complications with adjustment for age, sex, socioeconomic status, smoking, systolic blood pressure, BMI, total cholesterol, HbA1c, C-reactive protein, and serum uric acid.

**Findings:**

The prevalence of microvascular/macrovascular complications was higher in non-migrants than in migrants(nephropathy 15.3vs.9.7%; PAD 3.1%vs.1.3%; and CAD 15.8% vs. 5.0%). The differences persisted in the fully adjusted model: nephropathy [odds ratio, 2.12; 95% CI(1.46–3.08); PAD, 4.44(1.87–10.51); CAD 2.35(1.64–3.37)]. Non-migrant females had higher odds of nephropathy[2.14(1.34–3.43)], PAD[7.47(2.38–23.40)] and CAD [2.10(1.34–3.27)] compared to migrant females. Non-migrant males had higher odds of nephropathy[2.54(1.30–4.97)] and CAD[2.85(1.48–5.50)], but not PAD[1.81(0.32–10.29)],than their migrant peers.

**Interpretation:**

Macrovascular and renal microvascular complications were more prevalent in non-migrants than in migrant West Africans with IH. Further studies are needed to identify factors that increase the risk to aid preventive/treatment strategies.

## 1 Background

Worldwide, the prevalence of diabetes is increasing, but substantial variations exist between regions [1]. According to the International Diabetes Federation in 2021, about 24 million people in sub-Saharan Africa had diabetes; the number of people with diabetes is projected to more than double to 55 million by 2045, the highest projected increase anywhere in the world [1]. Diabetes increases the risk of vascular complications including microvascular disease (such as retinopathy, nephropathy, and neuropathy) and macrovascular disease (such as coronary artery disease (CAD), peripheral artery disease (PAD), and cerebrovascular disease) [[2], [3], [4]]. These vascular complications contribute to increased morbidity and early mortality [[2], [3], [4]].

Data from the multicenter Research on Obesity and Diabetes among African Migrants (RODAM) study have reported a high prevalence of microvascular and macrovascular complications in West Africans with type 2 diabetes (T2D) [5]. Additionally, the RODAM data showed that West Africans with T2D living in Africa had a higher prevalence of vascular complications compared with their migrant compatriots living in Europe [5]. The conventional cardiometabolic risk factors did not sufficiently explain these differences, especially for CAD and PAD [5].

Existing data show that the long-term macrovascular and microvascular dysfunction in diabetes may start manifesting at the intermediate hyperglycemia (IH) or prediabetes stage [6]. Once these vascular derangements begin, poor vascular care exacerbates progression and worsens the disease outcomes. In high-income settings like in Western Europe where there is greater access to healthcare, individuals with IH may be identified at an earlier stage, thereby allowing earlier intervention including vascular care, and halting disease progression [7]. Therefore, it could well be that the vascular functional derangements at the stage of IH may be a key contributor to the observed disparities in macrovascular and microvascular complication rates between West Africans living in Africa and their compatriots living in Europe. However, this has not been previously evaluated. This study aims to compare macrovascular and renal microvascular dysfunction among West Africans with IH living in West Africa and their migrant compatriots living in Europe.

## 2 Methods

### 2.1 Study design

The rationale, conceptual framework, design, and methodology of the RODAM study have been described in detail in a previous publication [8]. In brief, the RODAM study was conducted from 2012 to 2015 and included Ghanaian adults living in rural and urban Ghana as well as in three European cities (namely Amsterdam, Berlin, and London). Before the start of data collection, ethical approval was obtained from the respective ethics committees of the involved institutions in Ghana (School of Medical Sciences/Komfo Anokye Teaching Hospital Committee on Human Research, Publication & Ethical Review Board, ref. CHRPE/AP/200-12), UK (London School of Hygiene and Tropical Medicine Research Ethics Committee, ref. 6208), the Netherlands (Institutional Review Board of the Academic Medical Center, University of Amsterdam, ref. W12_062#12.17.0086) and Germany (Ethics Committee of Charité-Universitätsmedizin Berlin, ref. EA1/307/12). Informed written consent was also obtained from each participant before enrolment in the study. Data collection for the study was standardized across all sites.

The RODAM study included Ghanaians aged 25 years and above [8]. For the current analyses, participants aged 25–75 with IH were included (Fig. 1). This included data on 524 Ghanaians living in Ghana and 1439 Ghanaian migrants resident in Europe. IH was based on the American Diabetes Association (ADA) criteria and/or the World Health Organization's criteria for prediabetes. The ADA criterion was based on fasting plasma glucose (FPG) of 100 mg/dL (5.6 mmol/L) to 125 mg/dL (6.9 mmol/L) (impaired fasting glucose) or glycated hemoglobin (HbA1c) concentration of 5.7*-*6.4% (39*–*47 mmol/mol) in individuals not diagnosed as having diabetes based on either HbA1c or FPG [9, p. 20]. The WHO criterion was based on FPG of 6.0–6.9 mmol/L or HbA1c of 5.7–6.4% in individuals not diagnosed as having diabetes based on either HbA1c or FPG [9]. We used the term IH instead of prediabetes because of some controversies around the term prediabetes. Prediabetes suggests that these people go on and develop diabetes eventually, but recent studies have shown that for many that's not the case [10].

**Fig. 1:**
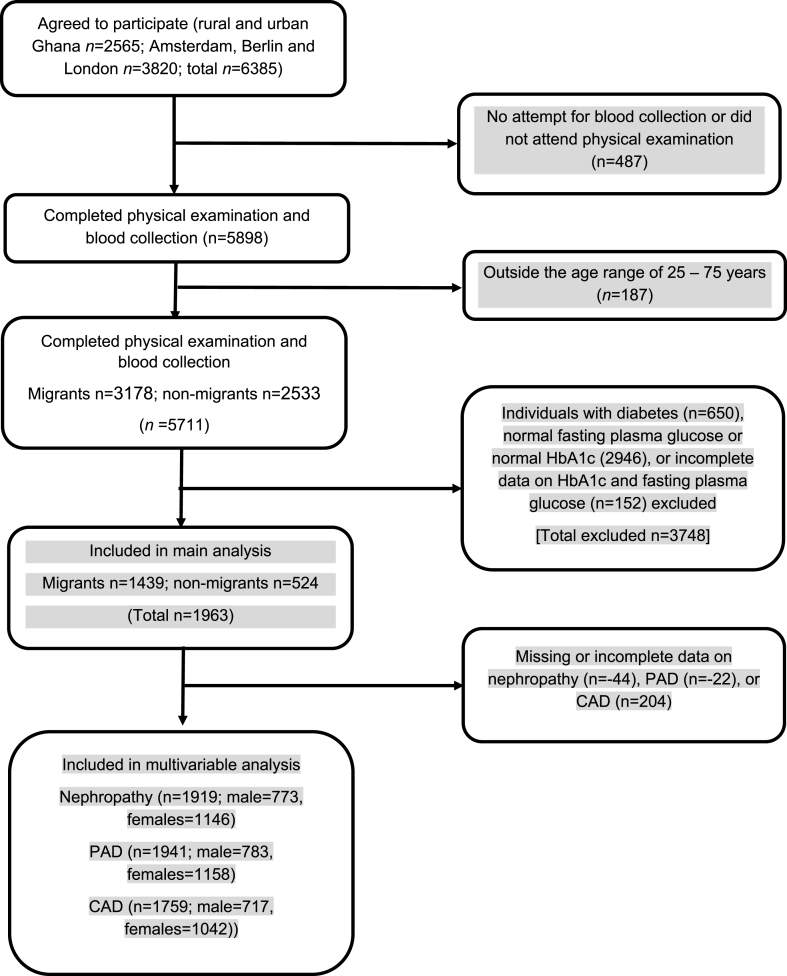
Flow chart of study design and inclusion in analyses Abbreviations: CAD = coronary artery disease; PAD = peripheral artery disease.

### 2.2 Assessments

The assessment of the baseline demographic, socioeconomic, and clinical measurements including the waist-to-hip ratio (WHR), and body mass index (BMI) have been described in detail elsewhere [8]. Blood pressure (BP) was measured three times using a validated semiautomated device (MicrolifeWatch BP home, Widnau, Switzerland), with appropriate-sized cuffs after at least 5 min rest while seated. The mean of the last two BP measurements was used for the analyses. Hypertension was defined as systolic BP (SBP) ≥140 mmHg and/or diastolic BP (DBP)≥90 mmHg, and/or being on antihypertensive medication treatment [11].

#### 2.2.1 Sample collection and biochemical analyses

Trained research assistants were used at all locations to take fasting (overnight fast of at least 10 h) venous blood samples. All samples were processed and divided into aliquots immediately after collection (within 1 h to a maximum of 3 h of the venipuncture), and then temporarily stored at the local research location at −20 °C. The separated samples were then transported to the local research centers’ laboratories, where they were checked, registered, and stored at −80 °C, before being shipped to their final destination for analysis. These samples included EDTA whole blood, heparin plasma, and serum. Concerning urine collection, participants were asked to bring the first early morning urine in a clean jar. One sample per participant was transported, together with the blood samples, to the respective local laboratory and stored at −80 °C.

Fasting glucose and lipid concentrations were determined using the ABX Pentra 400 chemistry analyzer (HORIBA ABX, Germany). Fasting plasma glucose concentration was measured using an enzymatic method (hexokinase). The concentration of total cholesterol was assessed by using colorimetric test kits. HbA1c was measured by high-performance liquid chromatography (TOSOH G8 HPLC analyzer). Serum creatinine concentration was determined by a kinetic colorimetric spectrophotometric isotope dilution mass spectrometry calibration method (Roche Diagnostics). The estimated glomerular filtration rate (eGFR) was calculated using the 2009 CKD-EPI (CKD Epidemiology Collaboration) creatinine equation and the severity of kidney disease categorized according to the 2012 KDIGO guidelines [12]. The concentration of urinary albumin (in μmol/L) was measured by an immunochemical turbidimetric method (Roche Diagnostics) and urinary creatinine concentration (in μmol/L) was measured by a kinetic spectrophotometric method (Roche Diagnostics). Serum uric acid concentration was measured using an enzymatic method (Trinder). The high-sensitivity C-reactive protein (CRP) concentration was measured in heparin plasma by a particle-enhanced immunoturbidimetric assay.

#### 2.2.2 Determination of microvascular and macrovascular complications

Ankle-brachial pressure index (ABI) measurements were performed by trained technicians in the supine position using a validated oscillometric device (Microlife WatchBP Office ABI, Switzerland) with appropriate-sized cuffs, after at least 10 min of supine rest. Systolic BP was measured twice in the right and left brachial arteries and twice in the right and left posterior tibial arteries. ABI was calculated by taking the highest arm systolic BP as the denominator, and the lowest ankle systolic BP as the numerator. The lowest of the left and right ABI measurements were used for analyses. ABI obtained by the oscillometric method using the Microlife WatchBP Office ABI has been shown to correlate well with ABI acquired by Doppler ultrasound with a 95% agreement between the two methods in diagnosing PAD [13]. PAD was defined as ABI ≤0.90 [14]. In defining normal ABI, an ABI >1.4 was excluded as it could be suggestive of non-compressible vessels [14]. CAD was based on the WHO Rose angina questionnaire [15]. The Rose questionnaire has a high specificity to detect CAD and is valuable for screening individuals in large-scale epidemiological surveys [16]. Albuminuria classifications were derived from the urinary albumin to creatinine ratio (ACR) and were as follows: A1, <3 mg/mmol (normal to mildly increased); A2, 3–30 mg/mmol (moderately increased) and A3, >30 mg/mmol (severely increased) [12]. Renal microvascular dysfunction was based on albuminuria, defined as ACR ≥3 mg/mmol [category ≥ A2]) according to the 2012 Kidney Disease Improving Global Outcomes guideline [12].

### 2.3 Statistical analyses

Data with a normal distribution were presented as mean ± standard deviation whereas those not normally distributed were presented as median (interquartile range). Categorical data were presented as frequencies (percentages). Differences in demographic, clinical, and vascular function between the migrant and non-migrant groups were assessed by chi-square test or two-sample independent sample *t*-test for categorical or continuous covariates, respectively. We first verified whether the association between IH and vascular complications varied by the site of residence (IH*site). A significant interaction was found for nephropathy, CAD, and PAD ((p < 0.001 for each). Multivariate logistic regression analyses were used to examine the associations between sites of residence in Ghana and Europe (independent variable) and microvascular/macrovascular dysfunction including nephropathy, CAD, and PAD (dependent variables), with adjustment for potential covariates. The minimal sufficient adjustment sets for estimating the direct effect of migration on microvascular/macrovascular dysfunction was determined by a directed acyclic graph (DAG) (DAG available at dagitty.net/mxklPBt) (Supplementary Fig. 1). Based on the DAG, the variables to be adjusted for were age, sex, socioeconomic status, dyslipidemia, glycemic control, hypertension, obesity, smoking, hyperuricemia, and low-grade inflammation. Aside from the conventional cardiometabolic risk factors, hyperuricemia and low-grade inflammation were included in the construction of the DAG because previous studies in this population show that they are key drivers of macrovascular/microvascular dysfunction [17,18]. Four models were used to examine the data: Model 1 was adjusted for age (and sex for the unstratified analyses); Model 2 was additionally adjusted for socioeconomic status (educational level); Model 3 was further adjusted for smoking; systolic blood pressure, BMI, total cholesterol, and HbA1c; Model 4 was further adjusted for CRP and serum uric acid. In a supplementary analysis, we further adjusted for cholesterol-lowering medications and antihypertensives. In a sensitivity analysis, associations were tested using IH based on the WHO definition only. The data were presented as odds ratios (OR) with their corresponding 95% confidence intervals (CI). A statistical test of significance was set at a p-value <0.05. Data were analyzed using the IBM SPSS version 25 for Windows.

## 3 Results

### 3.1 General characteristics

In the study population, the overall prevalence of IH was 34.4% (Fig. 1). The prevalence of IH was more than double in migrants than in non-migrants (45.3% versus 20.7%, p < 0.001).

Table 1 compares the baseline characteristics of migrant and non-migrant individuals with IH. Compared to the non-migrants, migrants were younger, more frequently males, more educated, had a higher proportion of current smokers, consumed more alcohol, and were less physically active. The mean BMI was 2.73 kg/m^2^ higher in migrants than in non-migrants. Mean systolic and diastolic blood pressures were respectively 6.93 mmHg and 4.12 mmHg higher in migrants than in non-migrants. The proportion of individuals with hypertension was higher in migrants than in non-migrants. Although the mean concentrations of HbA1c and serum uric acid were 2.95 mmol/mol and 25.38 mg/dL higher in migrants than in non-migrants, the mean concentrations of LDL-cholesterol and median CRP were 0.12 mmol/l and 1.71 mg/L higher in non-migrants than in migrants. Compared to non-migrants, migrants more frequently used blood pressure and cholesterol-lowering medications.

**Table 1:** Characteristics of Ghanaian migrants in Europe and non-migrant study participants with IH.

	Migrants^b^	Non-migrants^a^	*p-*value
Participants	*n* = 1439	*n* = 524	
Sex			0.001
Male	614(42.7%)	178(34.0%)	
Female	825(57.3%)	346(66.0%)	
Age (y)	48.01 ± 10.15	49.37 ± 11.10	0.010
Higher education	186(14.1%)	25(5.0%)	<0.001
Physical activity^c^			<0.001
Low level	339(31.5%)	149(29.9%)	
Moderate level	245(22.7%)	75(15.1%)	
High level	493(45.8%)	274(55.0%)	
BMI, kg/m^2^	29.33 ± 4.80	26.60 ± 5.49	<0.001
Waist-to-hip ratio	0.91 ± 0.07	0.91 ± 0.97	0.912
Current smokers (%)	55(4.2%)	5(1.0%)	0.001
Alcohol consumed, g/day	7.69 ± 19.83	1.48 ± 5.64	<0.001
Cholesterol-lowering medications (%)	106 (7.4%)	1 (0.2%)	<0.001
BP lowering medications (%)	455 (31.6%)	79 (15.1%)	<0.001
Systolic BP, mmHg	136.36 ± 17.87	129.43 ± 20.80	<0.001
Diastolic BP, mmHg	85.05 ± 11.12	80.93 ± 11.68	<0.001
Diagnosis of Hypertension	872(60.6%)	223(42.6%)	<0.001
Blood glucose, mmol/L	5.32 ± 0.64	5.53 ± 0.62	<0.001
HbA1c, mmol/mol	41.37 ± 3.67	38.42 ± 5.97	<0.001
Total cholesterol, mmol/l	5.15 ± 1.03	5.23 ± 1.24	0.148
LDL-cholesterol, mmol/l	3.32 ± 0.92	3.44 ± 1.06	0.014
C-reactive protein, mg/L	0.80(0.20–2.30)	1.10(0.30–4.10)	<0.001
Serum uric acid, mg/dl	338.55 ± 85.38	313.17 ± 83.63	<0.001

Definition of abbreviations: BMI = Body mass index; BP = Blood pressure; bpm = beats per minute; HbA1c = Glycosylated Hemoglobin; HDL = High-density lipoprotein; LDL = Low-density lipoprotein.

a Ghanaians resident in Ghana.

b Ghanaians living in Europe.

c Moderate - Any one of the following 3 criteria: ≥3 days of vigorous activity ≥20 min/day OR ≥5 days of moderate-intensity activity or walking ≥30 min/day OR >5 days of any combination of walking, moderate-intensity or vigorous intensity activities achieving a minimum of at least 600 MET-min/week. High - any one of the following 2 criteria: vigorous-intensity activity on ≥3 days and accumulating at least 1500 MET-minutes/week OR ≥7 days of any combination of walking, moderate-intensity, or vigorous-intensity activities achieving a minimum of at least 3000 MET-minutes/week. Low – not meeting criteria for moderate or high.

### 3.2 Macrovascular and microvascular dysfunction

Table 2 shows the differences in the microvascular and macrovascular functional status between the migrant and non-migrant groups. The mean eGFR of migrants was 3.3 mL/min/1.73 m^2^ lower in non-migrants than in migrants; the proportion of individuals with eGFR <60 mL/min/1.73 m^2^ (G3 – G5 categories) was 32.3% higher in non-migrants than in migrants. The proportions of individuals with albuminuria, PAD, and CAD were higher in non-migrants compared with migrants.

**Table 2:** Microvascular and Macrovascular Complications among Ghanaian migrants in Europe and non-migrants with IH.

	Migrants^b^	Non-migrants^a^	*p-*value
**Participants**	*n* = 1439	*n* = 524	
**CKD-EPI eGFR, mL/min/1.73 m**^**2**^	93.06 (±19.24)	89.76 (±19.80)	0.001
**CKD-EPI eGFR categories**			
**G1 and G2**	1351(97.1%)	492(94.1%)	0.019
**G3 – G5**	41(3.0%)	31 (6.0%)	0.019
**Albuminuria category**			
**A1, normal: <3 mg/mmol**	1289 (92.4.%)	462 (88.5%)	<0.001
**A2, moderate: 3–30 mg/mmol**	94 (6.7%)	54 (10.3%)	<0.001
**A3, severe: >30 mg/mmol**	12 (0.9%)	6 (1.1%)	<0.001
**Nephropathy**	135 (9.7%)	80 (15.3%)	<0.001
**PAD**	18 (1.3%)	16 (3.1%)	0.025
**CAD**	63 (5.0%)	79 (15.8%)	<0.001

Values for categorical variables are given as a number (percentage); for continuous variables, like mean (±standard deviation) or median (interquartile range).

Definition of abbreviations: ABI, Ankle Brachial Index; ACR, Albumin-creatinine ratio; CKD-EPI, Chronic Kidney Disease - Epidemiology Collaboration; eGFR, estimated glomerular filtration rate; PAD, Peripheral Artery Disease.

a Ghanaian residents living in Ghana.

b Ghanaian residents living in Europe.

Table 3 shows the odds for nephropathy, PAD, and CAD among non-migrants with IH compared with migrants. In models adjusted for age and sex, the odds of nephropathy, PAD, and CAD were higher in non-migrants than in their migrant compatriots. After further adjustment for socioeconomic status, smoking, hypertension, BMI, total cholesterol, HbA1c, CRP, and serum uric acid, the odds of nephropathy [adjusted odds ratio 2.12, 95% Cl, 1.46–3.08], PAD [4.44, 1.87–10.51] and CAD [2.35, 1.64–3.37] were higher in non-migrants than in migrants. Similar results were obtained when CRP >10 mg/L was excluded from the analyses. In the model further adjusted for cholesterol-lowering medications and antihypertensives (Supplementary Table 2), the odds of nephropathy [2.15, 1.47–3.15], PAD [5.24, 2.15–12.79] and CAD [2.55, 1.77–3.68] remained higher in non-migrants than in migrants.

**Table 3:** Multivariable logistic regression models for nephropathy, PAD, and CAD among Ghanaians with IH living in Ghana and Ghanaians living in Europe (reference = Ghanaians living in Europe). IH based on the ADA definition (n = 1963)^a^.

	OR (95% CI), p-value			
	Model 1	Model 2	Model 3	Model 4
*All Participants*				
Nephropathy (N = 1919)	1.56(1.16–2.11), 0.004	1.50(1.08–2.07), 0.015	2.08 (1.44–3.01), <0.001	2.12 (1.46–3.08), <0.001
PAD (N = 1941)	2.48(1.25–4.93), 0.009	3.13(1.47–6.66), 0.003	4.51 (1.92–10.61), 0.001	4.44 (1.87–10.51), 0.001
CAD (1759)	2.59(1.92–3.49), <0.001	2.38(1.74–3.25), <0.001	2.39 (1.68–3.42), <0.001	2.35 (1.64–3.37), <0.001
*Males only*				
Nephropathy (N = 773)	1.85(1.08–3.16), 0.025	1.69(0.95–2.99), 0.073	2.56 (1.31–4.98), 0.006	2.54 (1.30–4.97), 0.006
PAD (N = 783)	1.31(0.34–5.01), 0.689	1.85(0.45–7.67), 0.394	1.43 (0.26–7.92), 0.680	1.81 (0.32–10.29), 0.503
CAD (717)	3.33(1.96–5.66), <0.001	3.30(1.93–5.66), <0.001	2.76 (1.44–5.28), 0.002	2.85 (1.48–5.50), 0.002
*Females only*				
Nephropathy (N = 1146)	1.45(1.01–2.09), 0.043	1.38(0.93–2.04), 0.113	2.07 (1.31–3.27), 0.002	2.14 (1.34–3.43), 0.001
PAD (N = 1158)	3.27(1.42–7.55), 0.005	3.91(1.56–9.82), 0.004	7.28 (2.39–22.16), <0.001	7.47 (2.38–23.40), 0.001
CAD (N = 1042)	2.31(1.61–3.30), <0.001	2.00(1.37–2.93), <0.001	2.21 (1.42–3.43), <0.001	2.10 (1.34–3.27), 0.001

Definition of abbreviations: CAD = coronary artery disease; CI = Confidence interval; OR = odds ratio; PAD = Peripheral arterial disease.

Model 1 – adjusted for age and sex; model 2 – adjusted for age, sex, and socioeconomic status; model 3 – adjusted for age, sex, socioeconomic status, smoking; systolic blood pressure, body mass index, total cholesterol, and HbA1c; model 4 – adjusted for age, sex, socioeconomic status, smoking; systolic blood pressure, BMI, total cholesterol, HbA1c, CRP, and serum uric acid.

a IH based on HbA1c of 5.7%–6.5% or fasting plasma glucose of 5.6–6.9 mmol/L.

We examined whether the association between migration and vascular dysfunction differed by sex. As significant interaction effects (migrant status*sex) were found for at least one vascular measure (p < 0.001 for nephropathy, p < 0.001 for CAD, and p = 0.332 for PAD), we run sex-stratified analyses. In sex-stratified analyses, the odds of nephropathy [2.54, 1.30–4.97] and CAD [2.85, 1.48–5.50] were higher in non-migrant males, than in their migrant male counterparts, in the fully adjusted model. The odds of PAD in non-migrant males were not statistically significantly higher than in their migrant counterparts [1.81, 0.32–10.29]. In females, the odds of nephropathy [2.14, 1.34–3.43], CAD [2.10, 1.34–3.27], and PAD [7.47, 2.38–23.40] were higher in non-migrants than in migrants, after adjustment for all the potential covariates. Similar observations were made when IH was defined based on the WHO guidelines instead of the ADA guidelines (Supplementary Table 1).

## 4 Discussion

### 4.1 Key findings

The prevalence of IH was over twice as high in migrants compared with non-migrants. Among individuals with IH, the odds of macrovascular and renal microvascular complications were higher in non-migrants than in migrants. These differences in macrovascular and renal microvascular complications persisted after adjustment for a wide range of covariates including the conventional cardiovascular risk factors, serum uric acid concentration, and low-grade inflammation. Except for PAD in males, similar observations were made in sex-stratified analyses.

### 4.2 Discussion of key findings

In this study, we observed a higher prevalence of IH among migrants compared with non-migrants. Although we found no prior study comparing IH in a migrant group and their non-migrant compatriots, our findings are in line with the existing evidence linking migration to cardiometabolic disease including type 2 diabetes [19]. Post-migration factors likely to increase the likelihood of IH include altered dietary habits (for example adoption of a Western diet characterized by high consumption of red meat, processed meat, sweets, and high-fat dairy products), decreased physical activity, and increased psychosocial stress, which are key drivers of obesity and impaired fasting glucose [19].

The prevalence of IH among the migrant group (45.3%) is higher than those reported in European ancestry populations including the Netherlands (15%) [20], the United States (34.4%) [21], and the United Kingdom (35.3%) [22]. This is in agreement with previous reports of higher incidence rates of IH among non-European migrants, compared with the high-income host population [23]. Potential contributory factors include factors limiting migrants from engaging in healthy lifestyles. For example, migrants frequently congregate in deprived neighborhoods with limited access to healthy food options, a safe walking environment for physical exercise, and limited social support systems which increase their psychosocial stress [19]. The high prevalence of IH among migrants is of clinical and public health significance because individuals with IH may have a higher cardiometabolic disease risk including T2D [10].

We observed that non-migrants with IH who in general had better modifiable cardiometabolic risk profiles (including being more physically active, less frequently smokers, consuming less alcohol, and having lower mean BMI, systolic blood pressures, HbA1c concentration, and serum uric acid concentrations), were more likely to have macrovascular and renal microvascular complications compared with their migrant compatriots. The key modifiable risk factors favoring migrants (concerning the prevalence of macrovascular/microvascular dysfunction) were lower CRP and LDL cholesterol concentrations (and a higher proportion of individuals on cholesterol-lowering medications). Given the important roles of smoking, physical inactivity, hypertension, obesity, hyperuricemia, and poor glycemic control in the development and progression of macrovascular and renal microvascular complications, it is uncertain whether higher concentrations of LDL-cholesterol and CRP alone can tip the balance against the non-migrant group. In particular, smoking and poorer glycemic control are known to play greater roles than hyperlipidemia in the development of PAD [24] while elevated systolic blood pressure and higher HbA1c are robust independent risk factors for renal microvascular dysfunction. However, the proportion of smokers in this population was remarkably low, and the study population had glycemic levels below the threshold for diabetes.

Notwithstanding, the roles of elevated CRP and LDL-cholesterol concentrations in driving atherosclerotic macrovascular complications as well as renal microvascular dysfunction cannot be underestimated. In the general population without known cardiovascular disease, elevated CRP concentration is associated with an increased risk of vascular dysfunction [25]. Previously, our team reported that within the CRP range of <10 mg/L, elevated CRP is significantly associated with PAD and renal microvascular dysfunction in West Africans without diabetes [18]. Although the reasons for the elevated CRP concentration in the non-migrant group remain unclear, a higher prevalence of chronic or recurrent infection in non-migrants could a potential reason. Bacterial infections, which may contribute to elevated CRP concentration, are a potential cause of PAD [26] and CAD [27]. Targeting causes of elevated CRP concentrations could be valuable in reducing the likelihood of macrovascular and renal microvascular complications in individuals with IH.

Elevated total cholesterol and LDL cholesterol concentrations are known to pose a lifelong risk of atherosclerotic cardiovascular disease [28]. Therefore, the relatively lower cholesterol concentrations in the migrant group might have offered them some vascular protection. In this study population, the low-LDL cholesterol concentration could be due to multiple factors including more frequent use of cholesterol-lowering therapy including statins. Migrants with or without vascular complications were more likely to be treated with anticholesterol (and antihypertensive medications((Supplementary Table 3), which could have contributed to the decreased likelihood of vascular complications. Statins may improve vascular health via a mechanism independent of cholesterol-lowering [29]; these benefits are known to be pronounced in individuals with diabetes. Like anticholesterol medications, antihypertensive therapy is known to protect against vascular complications in individuals with IH. While non-migrants had a lower proportion of individuals on antihypertensives, this could also reflect the lower proportion of non-migrants with hypertension.

Sex-stratified analyses yielded similar results, except for PAD in males, where the odds of PAD in non-migrant males were not statistically significantly higher than in their migrant counterparts. However, the direction of association between migrant status and the likelihood of PAD in males was similar to that in females, as well as for CAD and renal microvascular dysfunction. Therefore, our observation might just be a matter of sample size and power to detect significant differences. Also likely to be related to power limitations, the odds ratio for PAD in non-migrant females was remarkably high with a wider 95% confidence interval. This is likely as the biological determinants of PAD [24] do not vary substantially between males and females.

Our study also shows that after adjustment for the above-discussed cardiometabolic risk factors, the likelihood of macrovascular and renal microvascular disease was still higher in non-migrants than in migrants. Given the inability of the measured biological and socioeconomic variables to fully explain the higher likelihood of vascular complications in non-migrants (who live in the low-resource setting) compared with migrants (who live in high-resource settings), other factors including the differences in the quality of healthcare delivery, as well as differences in health-seeking attitudes and behaviors between the migrants and non-migrant groups, may be important. For example, individuals living in high-resource settings often have better access to healthcare and preventive services than their non-migrant compatriots living in low-income settings, thereby aiding in earlier identification of IH and commencement of vascular care interventions including targeting lower blood pressure and blood cholesterol cut-off values. Future studies could look at the predictive roles of screening on IH and preventive care and healthcare processes, and their effects on the control of vascular risk factors as well as the development of vascular complications in individuals with IH.

#### 4.2.1 Strengths and limitations

To the best of our knowledge, our study is the first to report on a comparison of microvascular and macrovascular dysfunction among a homogeneous group of individuals with IH living in different geographical/resource settings. Key strengths of our study are that we used a relatively homogenous multi-centered study population of people of West African origin and well-standardized study protocols across the various study sites. Additionally, we eliminated the limitation of intra-laboratory variability by using the same standard operating procedures in the same laboratory for running all samples across all sites. Furthermore, we adjusted for a wide range of conventional cardiometabolic risk factors as well as other biomarkers implicated in vascular injury including serum uric acid and CRP. Our study has limitations. First, the cross-sectional design limits us from claiming causality. Secondly, our definition of IH did not include 2-h plasma glucose which was not measured due to feasibility. Thirdly, we assessed CAD via the Rose Angina Questionnaire, instead of invasive coronary angiography, the gold standard. Although the Rose Angina Questionnaire has moderate sensitivity, it has a high specificity to detect CAD and is valuable for screening individuals at risk of CAD in large-scale epidemiological surveys [16]. Fourthly, we did not assess microvascular dysfunction in other circulations including the retinal and neural circulations, and neither did we assess dysfunctions in the cerebral, and coronary circulation. However, albuminuria which assesses nephropathy may also reflect generalized endothelial and microvascular dysfunction [[30], [31]]. Last, the observed sex differences in PAD may reflect limited power to detect significant differences for PAD.

## 5 Conclusion

West Africans with IH had a high prevalence of macrovascular and renal microvascular complications. The odds of macrovascular and renal microvascular complications were higher in non-migrants than in migrants with IH, after adjustment for a wide range of cardiovascular risk factors. Similar observations were made in both males and females in sex-stratified analyses. Monitoring and treatment of macrovascular and renal microvascular complications in these individuals may be important, regardless of whether they progress to T2D or not. Further studies including those aimed at identifying factors that increase the risk of macrovascular and renal microvascular complications in non-migrant West Africans with IH are needed to aid prevention and treatment strategies.

## Author contribution statement

Emmanuel Bannerman-Williams, Charles F Hayfron-Benjamin, Yacoba Atiase: Analyzed and interpreted the data; Wrote the paper.

Charles Agyemang, Erik Beune, Karlijn Meeks: Conceived and designed the experiments; Performed the experiments; Wrote the paper.

Bert-Jan van den Born, Silver Bahendeka, Kerstin Klipstein-Grobusch, Juliet Addo, Frank Mockenhaupt, Matthias B. Schulze: Conceived and designed the experiments; Wrote the paper.

## 6 Data sharing

The datasets created and/or analyzed during the current study are available from the RODAM Executive Board upon reasonable request.

## Funding

This work was supported by the 10.13039/100011102European Commission under the Framework Programme (Grant Number: 278901). The study sponsor was not involved in the design of the study; the collection, analysis, and interpretation of data; writing the report; nor the decision to submit the report for publication. Karlijn Meeks. is supported by the Intramural Research Program of the 10.13039/100000002National Institutes of Health in the Center for Research on Genomics and Global Health (CRGGH). The CRGGH is supported by the 10.13039/100000051National Human Genome Research Institute, the 10.13039/100000062National Institute of Diabetes and Digestive and Kidney Diseases, the Center for Information Technology, and the Office of the Director at the 10.13039/100000002National Institutes of Health (1ZIAHG200362).

## 7 Patient consent for publication

Not required.

## Declaration of competing interest

The authors declare that they have no known competing financial interests or personal relationships that could have appeared to influence the work reported in this paper.

## Figures and Tables

**figs1:**
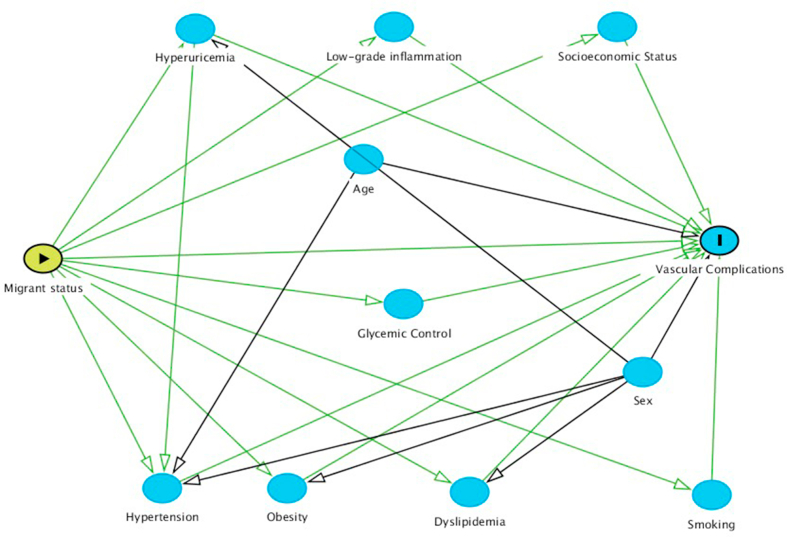


## Nonstandard abbreviations and acronyms

ABI: ankle-brachial pressure index
BMI: body mass index
BP: blood pressure
CAD: coronary artery disease
CI: confidence interval
IH: Intermediate Hyperglycemia
OR: odds ratio
PAD: peripheral artery disease
RODAM: Research on Obesity and Diabetes among African Migrants
T2D: type 2 diabetes
