# Supplementary material for: Macrovascular and renal microvascular complications in West Africans with intermediate hyperglycemia living in West Africa and Europe: The RODAM study
Source: Heliyon. 2023 Aug 20;9(8):e19334. doi: 10.1016/j.heliyon.2023.e19334 (PMC10469941; doi:10.1016/j.heliyon.2023.e19334)
Supplement: Multimedia component 1 [file mmc1.docx]

Supplementary Table 1: Multivariable logistic regression models for nephropathy, PAD, and CAD among Ghanaians with IH living in Ghana and Ghanaians living in Europe (reference = Ghanaians living in Europe). *IH based on WHO definitions (n=1686)**.

|  | OR (95% ci), p-value | | | |
| --- | --- | --- | --- | --- |
|  | Model 1 | Model 2 | Model 3 | Model 4 |
| *All Participants* |  |  |  |  |
| Nephropathy (N=1646) | 1.65(1.18-2.31), 0.004 | 1.64(1.15-2.34), 0.007 | 2.16 (1.45-3.22), <0.001 | 2.20 (1.46-3.31), <0.001 |
| PAD (1669) | **2.65 (1.27-5.53), 0.010** | 3.50 (1.55-7.90), 0.003 | 5.22 (2.10-13.01), <0.001 | 5.06 (2.00-12.85), 0.001 |
| CAD (N=1510) | 2.29 (1.63-3.21), <0.001 | 2.03 (1.42-2.90), <0.001 | 2.22 (1.50-3.28), <0.001 | 2.18 (1.46-3.24), <0.001 |
|  |  |  |  |  |
| *Males only* |  |  |  |  |
| Nephropathy (N=639) | 1.76(0.94-3.30), 0.076 | 1.83(0.95-3.53), 0.072 | 2.47 (1.16-5.23), 0.018 | 2.41 (1.11-5.22), 0.025 |
| PAD (N=650) | 1.34 (0.27-6.54), 0.719 | 2.28 (0.43-12.10), 0.331 | 1.79 (0.24-13.11), 0.567 | 2.23 (0.29-17.24), 0.444 |
| CAD (N=593) | 2.44 (1.27-4.67), 0.007 | 2.38 (1.22-4.64), 0.011 | 2.41 (1.11-5.21), 0.026 | 2.66 (1.21-5.81), 0.014 |
|  |  |  |  |  |
| *Females only* |  |  |  |  |
| Nephropathy (N=1007) | 1.60(1.07-2.38), 0.022 | 1.53(1.00-2.35), 0.051 | 2.19 (1.34-3.57), 0.002 | 2.24 (1.36-3.70), 0.002 |
| PAD (1019) | 3.36 (1.41-8.04), 0.006 | 3.94 (1.51-10.29), 0.005 | 6.87 (2.20-21.45), 0.001 | 6.81 (2.12-21.85), 0.001 |
| CAD (N=917) | 2.23 (1.50-3.32), <0.001 | 1.89 (1.24-2.87), 0.003 | 2.16 (1.36-3.45), 0.001 | 2.07 (1.29-3.33), 0.003 |

Definition of abbreviations: CAD = Coronary artery disease; CI = Confidence interval; OR = odds ratio; PAD = Peripheral arterial disease.

Model 1 – adjusted for age and sex; model 2 – adjusted for age, sex, and socioeconomic status; model 3 – adjusted for age, sex, socioeconomic status, smoking; systolic blood pressure, body mass index, total cholesterol, and HbA1; model 4 – adjusted for age, sex, socioeconomic status, smoking; systolic blood pressure, BMI, total cholesterol, HbA1, CRP, and serum uric acid.

* IH based on HbA1c of 5.7% – 6.5% and fasting plasma glucose of 6.0 - 6.9 mmol/L.

Supplementary Table 2: Multivariable logistic regression models additionally adjusted for cholesterol-lowering and antihypertensive medications. Models for nephropathy, PAD, and CAD among Ghanaians with IH living in Ghana and Ghanaians living in Europe (reference = Ghanaians living in Europe).

|  | OR (95% ci), p-value |
| --- | --- |
| *All Participants* |  |
| Nephropathy (N=1646) | 2.15 (1.47-3.15), <0.001 |
| PAD (1669) | 5.24 (2.15-12.79), <0.001 |
| CAD (N=1510) | 2.55 (1.77-3.68), <0.001 |
|  |  |
| *Males only* |  |
| Nephropathy (N=639) | 2.52 (1.27-5.02), 0.008 |
| PAD (N=650) | 1.83 (0.32-10.57), 0.501 |
| CAD (N=593) | 3.20 (1.61-6.35), 0.001 |
|  |  |
| *Females only* |  |
| Nephropathy (N=1007) | 2.23 (1.39-3.59), 0.001 |
| PAD (1019) | 10.07 (2.93-34.58), <0.001 |
| CAD (N=917) | 2.22 (1.41-3.48), 0.001 |

Definition of abbreviations: CAD = Coronary artery disease; CI = Confidence interval; CVD = cardiovascular disease; OR = odds ratio; PAD = Peripheral arterial disease.

Model adjusted for age, sex, socioeconomic status, smoking; systolic blood pressure, body mass index, total cholesterol, HbA1; c-reactive protein, serum uric acid, cholesterol-lowering, and antihypertensive medications.

Supplementary Table 3: Microvascular and Macrovascular Complications among migrants and non-migrants with IH stratified by ant cholesterol and antihypertensive therapy.

|  | Nephropathy | | | CAD | | | PAD | | |
| --- | --- | --- | --- | --- | --- | --- | --- | --- | --- |
|  | No | Yes | *p-value* | No | Yes | *p-value* | No | Yes | *p-value* |
| **ALL PARTICIPANTS** |  |  |  |  |  |  |  |  |  |
| Cholesterol-lowering medications (%) | 91 (5.3%) | 12 (5.6%) | 0.490 | 79 (5.1%) | 6.8% | 0.191 | 103 (5.4%) | 1 (2.9%) | 0.621 |
| BP lowering medications (%) | 440 (25.8%) | 82 (38.1%) | <0.001 | 410 (26.4%) | 67 (32.5%) | 0.040 | 512 (26.8%) | 15 (44.1%) | 0.024 |
|  |  |  |  |  |  |  |  |  |  |
| **MIGRANTS** |  |  |  |  |  |  |  |  |  |
| Cholesterol-lowering medications (%) | 90 (7.1%) | 12 (8.9%) | 0.275 | 78 (6.8%) | 14 (13.2%) | 0.018 | 102 (7.3%) | 1 (5.6%) | 0.969 |
| BP lowering medications (%) | 387 (30.7%) | 56 (41.5%) | 0.008 | 351 (30.4%) | 47 (44.3%) | 0.003 | 444 (31.6%) | 6 (33.3%) | 0.528 |
|  |  |  |  |  |  |  |  |  |  |
| **NON-MIGRANTS** |  |  |  |  |  |  |  |  |  |
| Cholesterol-lowering medications (%) | 1 (0.2%) | 0 (0.0%) | 0.847 | 1 (0.3%) | 0 (0.0%) | 0.800 | 1 (0.2%) | 0 (0.0%) | 0.448 |
| BP lowering medications (%) | 53 (12.0%) | 26 (32.5%) | <0.001 | 59 (14.8%) | 20 (20.0%) | 0.129 | 68 (13.5%) | 9 (56.3%) | <0.001 |

Supplementary Figure 1: Directed acyclic graph – available at [dagitty.net/mxklPBt](http://dagitty.net/mxklPBt)

*Minimal sufficient adjustment sets for estimating the direct effect of migrant status on vascular complications: Age, Dyslipidemia, Glycemic Control, Hypertension, Hyperuricemia, Low-grade inflammation, Obesity, Sex, Smoking, Socioeconomic Status*
